# Synoptic tool for reporting of hematological and lymphoid neoplasms based on World Health Organization classification and College of American Pathologists checklist

**DOI:** 10.1186/1471-2407-7-144

**Published:** 2007-07-31

**Authors:** Sambit K Mohanty, Anthony L Piccoli, Lisa J Devine, Ashokkumar A Patel, Gross C William, Sharon B Winters, Michael J Becich, Anil V Parwani

**Affiliations:** 1 Department of Biomedical Informatics, University of Pittsburgh School of Medicine, Pittsburgh, PA, USA; 2 Department of Pathology, University of Pittsburgh School of Medicine, Pittsburgh, PA, USA

## Abstract

**Background:**

Synoptic reporting, either as part of the pathology report or replacing some free text component incorporates standardized data elements in the form of checklists for pathology reporting. This ensures the pathologists make note of these findings in their reports, thereby improving the quality and uniformity of information in the pathology reports.

**Methods:**

The purpose of this project is to develop the entire set of elements in the synoptic templates or "worksheets" for hematologic and lymphoid neoplasms using the World Health Organization (WHO) Classification and the College of American Pathologists (CAP) Cancer Checklists. The CAP checklists' content was supplemented with the most updated classification scheme (WHO classification), specimen details, staging as well as information on various ancillary techniques such as cytochemical studies, immunophenotyping, cytogenetics including Fluorescent In-situ Hybridization (FISH) studies and genotyping. We have used a digital synoptic reporting system as part of an existing laboratory information system (LIS), CoPathPlus, from Cerner DHT, Inc. The synoptic elements are presented as discrete data points, so that a data element such as tumor type is assigned from the synoptic value dictionary under the value of tumor type, allowing the user to search for just those cases that have that value point populated.

**Results:**

These synoptic worksheets are implemented for use in our LIS. The data is stored as discrete data elements appear as an accession summary within the final pathology report. In addition, the synoptic data can be exported to research databases for linking pathological details on banked tissues.

**Conclusion:**

Synoptic reporting provides a structured method for entering the diagnostic as well as prognostic information for a particular pathology specimen or sample, thereby reducing transcription services and reducing specimen turnaround time. Furthermore, it provides accurate and consistent diagnostic information dictated by pathologists as a basis for appropriate therapeutic modalities. Using synoptic reports, consistent data elements with minimized typographical and transcription errors can be generated and placed in the LIS relational database, enabling quicker access to desired information and improved communication for appropriate cancer management. The templates will also eventually serve as a conduit for capturing and storing data in the virtual biorepository for translational research. Such uniformity of data lends itself to subsequent ease of data viewing and extraction, as demonstrated by rapid production of standardized, high-quality data from the hemopoietic and lymphoid neoplasm specimens.

## Background

The rise of molecular and translational medicine attempting to more directly connect basic science and clinical research to patient care emphasizes the development of efficient and sophisticated technologies in the laboratory environment to extract maximum information from various diagnostic models. The surgical pathology report is one such source that carries a wealth of information, and serves as an efficient vehicle conveying the morphologist's view to the clinicians that ultimately gets reflected in clinical evaluation and further management of the patient [1]. Therefore, clarity, accuracy and thoroughness are the three important aspects of a report; however a substantial amount of variability in format and context exists [1]. Over the last decade, a considerable amount of effort by morphologists, researchers and informaticians has been devoted to development of mechanisms in the form of templates, checklists, and tables to make the pathology reports more useful [1-7]. Such mechanisms are designed to ensure consistency in the content of reports regardless of the institution of origin. The synoptic reporting system is one such innovation, with the goal to provide a structured and pre-formatted method for entering clinically and morphologically relevant details of surgical specimens, where the resulting information is then searchable as discrete elements rather than by cumbersome natural language processes, and selected data can be passed to a repository via a results interface. It offers an "online diagnosis worksheet" that is easily learned and deployed, and may encourage some pathologists to enter the diagnostic information themselves, thereby reducing transcription services and reducing specimen turnaround time [3,4,7,8]. It provides templated data entry with uniformity and accuracy, including diagnostic content from both gross and microscopic examination. This templated presentation of data on reports prioritizes diagnostic information and streamlines access by clinicians. Gross and microscopic examination of surgical specimens, particularly large resections yields comprehensive information with implications for ongoing and future medical and oncology care. These distinct data elements can be captured in this template and transmitted electronically to the data base systems to enhance basic science, clinical and translational cancer research. The College of American Pathologists Cancer Protocols and Checklists were created with the objective of improving the quality and uniformity of information in pathology reports that eventually culminated in patient care and management [6,9,10].

## Methods

### Study Design

The purpose of this project was to develop the entire set of elements in the synoptic templates for hematological and lymphoid neoplasms (bone marrow malignancies, Non-Hodgkin, Hodgkin and gastrointestinal lymphoma) using WHO Classification and CAP Checklists [6,11-14]. The CAP checklists were supplemented with the most updated classification schema (WHO classification), specimen details, staging as well as information on various ancillary techniques such as cytochemical studies, immunophenotyping, cytogenetics including FISH studies and genotyping.

### Technology

We have used a digital synoptic reporting module integrated into an existing laboratory information system, CoPathPlus (v2.5.1.83), owned and developed by Cerner DHT, Inc. [15]. The LIS provides a Windows-based user interface organized into workflow-related "activities", and is built on a relational database platform (Sybase). The module includes predefined synoptic worksheets based on CAP checklists for bone marrow malignancies, Non-Hodgkin's, Hodgkin and Gastrointestinal Lymphomas, including WHO disease classifications, which we then updated per current schema and other ancillary elements as noted above. The data elements are presented under logical categories or headers and captured as discrete values; e.g., data element for WHO classification ''lymphoplasmacytic lymphoma' exists in the synoptic value dictionary as a discrete value, allowing the user to search for just those cases with that value point populated.

There are 4 distinct components within the synoptic reporting system that are as follows (Fig. 1, 2, 3, 4, 5, 6, 7, 8, 9):

**Figure 1:**
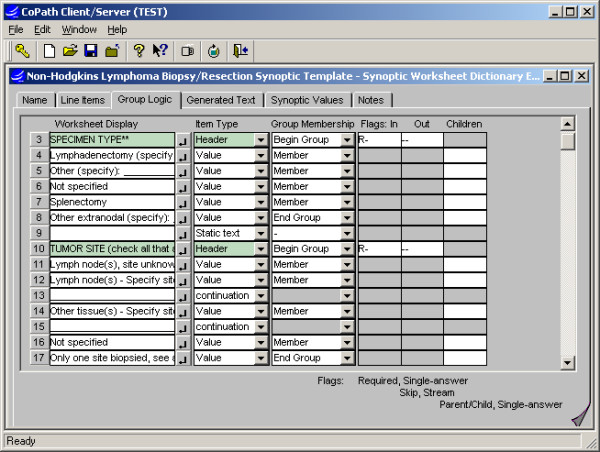
Synoptic Worksheet dictionary interface: The template for entering structured data is assembled from defined categories and values, or "questions" and "answers", which can be inserted as related groupings. Additional logic may then be applied to groupings, e.g. to require an entry or allow for multiple entries. The same tool is used for subsequent editing.

**Figure 2:**
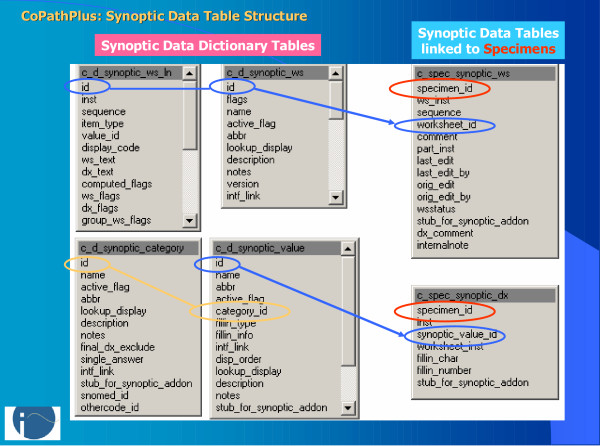
CoPathPlus synoptic table structure: Tables on the left contain the defined synoptic templates, and structured categories and values applied to templates. Tables on the right contain template-level data and values selected for distinct specimens, with links between the tables indicated by colored lines.

**Figure 3:**
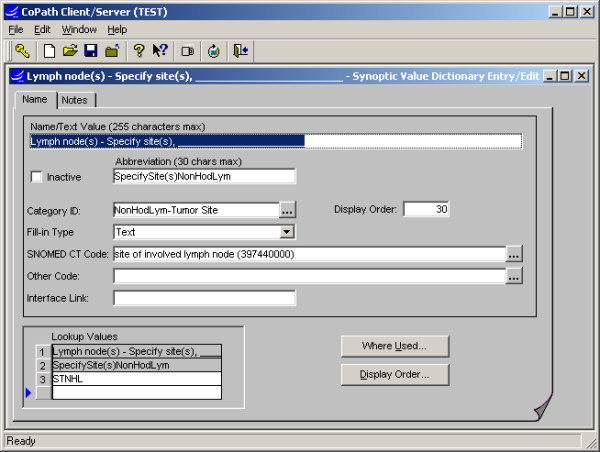
Synoptic Value dictionary interface: The Values to be applied to worksheets are defined or edited and attributes configured, e.g. to allow numeric or text fill-ins or link SNOMED CT code.

**Figure 4:**
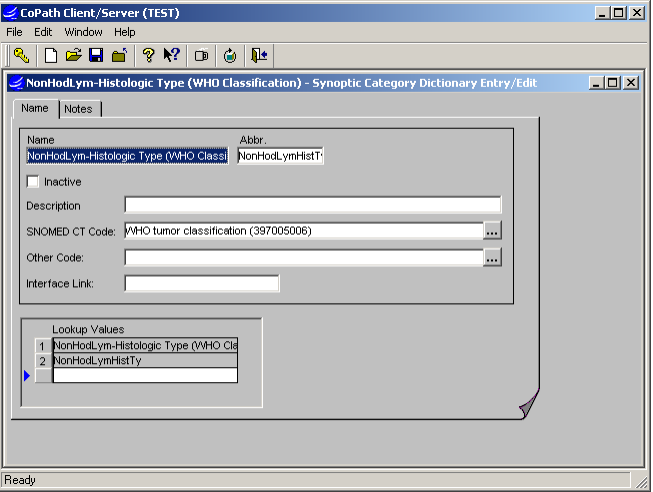
Synoptic Category dictionary interface: Categories are defined to organize the synoptic values and function in adding values to templates in groupings (see Fig. 1). They may also be accessed in constructing data retrievals or lookups.

**Figure 5:**
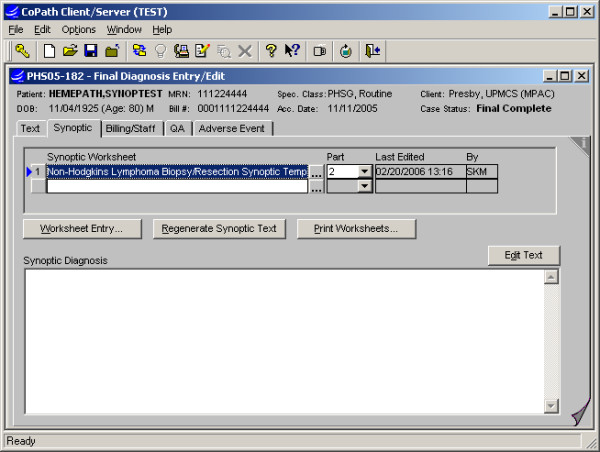
Final Diagnosis Entry/Edit activity: This application item is used for primary entry of final diagnosis and related text, either by pathologists or clerical staff, and access to synoptic worksheet entry is integrated as a second tab. A "blank" worksheet may be previously added to the case or defaulted based on specimen type. The first button is used to open the worksheet for entry or editing.

**Figure 6:**
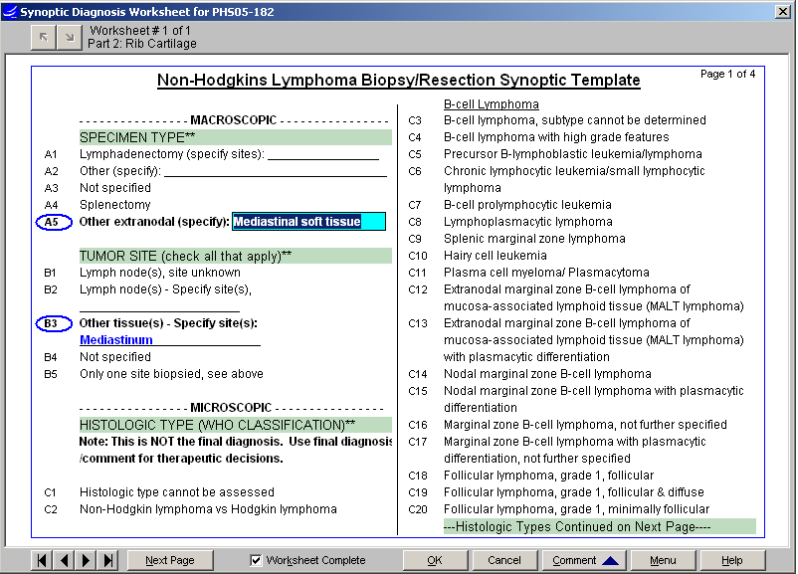
Synoptic Worksheet entry interface, Non-Hodgkin's Lymphoma: The template is presented in multiple pages, as lists of related values with logical headers. Selected values are circled in blue. If a selected value includes a fill-in, a light blue text box displays for typed entry (see A5 above) and then displays in bold blue (see B3). Other optional conventions shown include an off-colored band for the header and a double-asterisk (**) to denote groups which require an entry.

**Figure 7:**
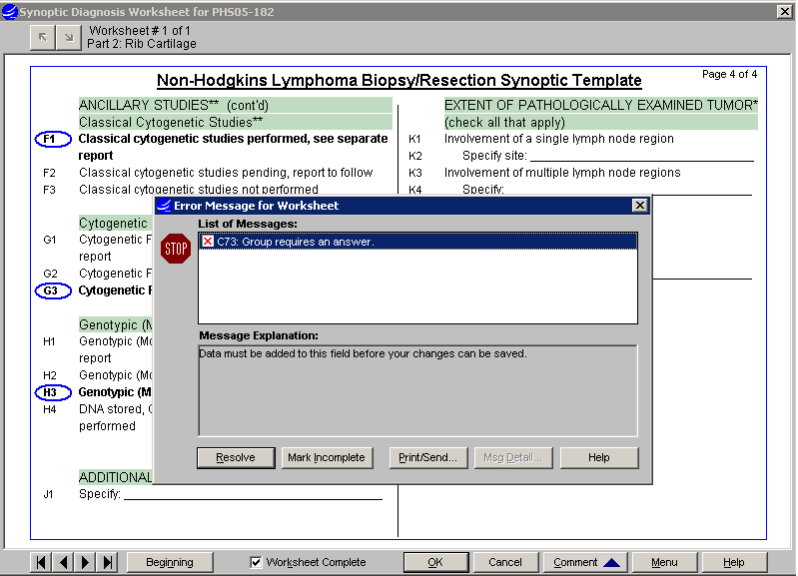
Synoptic Worksheet entry interface, Non-Hodgkin's Lymphoma: After selections are made for all categories, the template is marked "Worksheet Complete". Validation logic will generate messages to user, e.g. for a required category having no selection, and a "Resolve" response moves user to the relevant section. A worksheet generates no text in the report until successfully completed.

**Figure 8:**
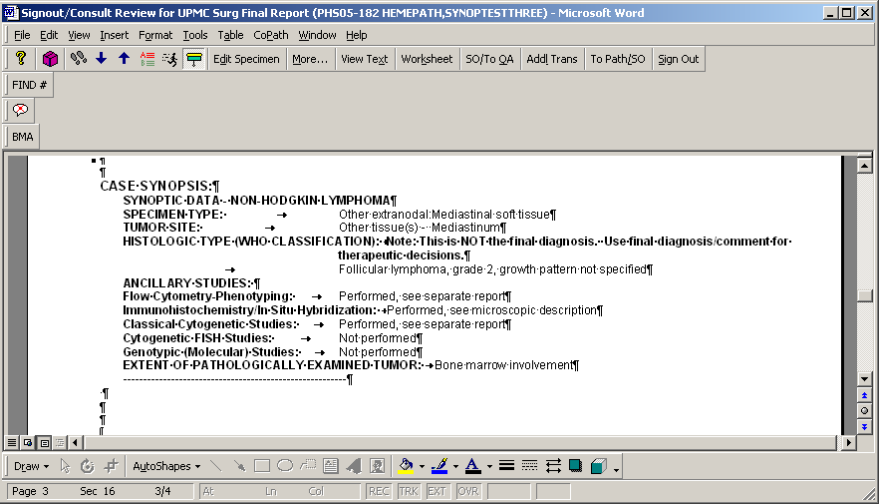
Final Report Sign-out: A completed synoptic worksheet will auto-generate text values for all selected items to the final report, in a distinct section, with categories and values in opposing columns. The synoptic text may not be edited directly. The "Worksheet" button will access the synoptic entry screen, to allow pathologist to complete or otherwise edit selections, and text is then updated dynamically. NOTE: The phrase 'This is NOT the final diagnosis...' above the synoptic Histologic Type is intended to alert the clinician that more extensive information regarding the diagnosis is often included in the narrative text section of the report, along with commentary on the diagnosis, and that a quick scan of the synoptic report content may not be adequate to appreciate complexities of the case.

**Figure 9:**
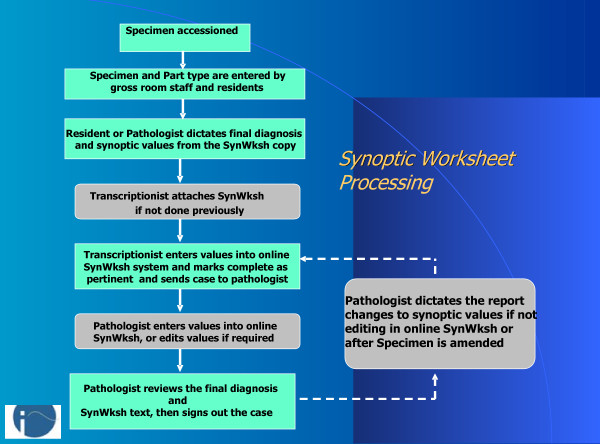
Workflow for Synoptic Worksheet processing: Multiple steps contributing to synoptic data output occur during processing of a case. Grey boxes represent optional or alternate steps used in certain cases, e.g. where pathologist defers to dictation of worksheet changes.

#### Dictionaries (Fig. 1, 2, 3, 4)

##### Synoptic Category Dictionary

The Synoptic Categories are defined and associated with logical headers or "questions" in the checklist, e.g. "specimen type" or "WHO classicification", to which synoptic values are then assigned. Categories are used to add selected groups of values to a worksheet and provide a default header for the groups, and may also be used later to facilitate queries.

##### Synoptic Value Dictionary

a Synoptic Value entry is created for each distinct item to be selected on a Synoptic Worksheet. The values can be designated to include a fill-in (text or numeric type) in the Synoptic Value dictionary, if appropriate. SNOMED CT codes may also be associated with values in this dictionary, to support query activity or export to another database at a later time.

##### Synoptic Worksheet Dictionary

Synoptic "worksheets" (templates) are defined and assembled in the Synoptic Worksheet dictionary. The key items on a worksheet are defined in the Synoptic Value and Synoptic Category Dictionaries, as above. For example, The Non-Hodgkin's Lymphoma Synoptic worksheet contains a category labeled "Specimen Type" and under this category are multiple Synoptic Values that can be selected to indicate the type of specimen that was submitted for evaluation. Within the Synoptic Worksheet, categories or groups of values can be set to require a selection within the group; to allow single or multiple selections within the group; and to omit printing of the category header in the generated text if no value is selected. Attributes of the text generated to reports for both group headers and values can also be designated in the Synoptic Worksheet Dictionary.

##### Synoptic Worksheet Group dictionary

Worksheets can optionally be associated with synoptic worksheet "groups," which may be used for data search purposes.

Part Type dictionary – allows one or more default synoptic worksheets to be specified per part type assigned to a specimen.

#### Specimen data entry and text generation (Fig. 5, 6, 7, 8)

In the Final Diagnosis Entry/Edit and Electronic Sign-out activities, the windows can be painted to enable synoptic data entry/editing in line with editing of other diagnostic text in the report. The worksheets may be accessed and completed directly by pathologists or training staff, or dictated and completed by transcription staff. Validation logic based on definition of worksheets generates warnings to staff, e.g. for required groups missing a selected value or fill-ins with inconsistent formatting. After the worksheets are filled out and completed online, the system automatically generates diagnosis text from them based on specifications in the Synoptic Worksheet definition. The generated text can be directed to the Final Diagnosis text field or to a separate text field. Text generated onto the report is protected from editing via the word processor, to prevent changes being made to the generated text without corresponding changes to the synoptic values that were the source of that text. Free text comments can be included in the text field, either above or below the protected text.

#### Results interface

An HL-7 results interface can optionally be configured to transmit discrete synoptic data elements via "Z segment" extensions, along with the text-based HL-7 results.

#### Data search and management reports

A data search capability is provided via the "Infomaker wizard" tool, allowing detailed searches of the discrete synoptic data fields in combination with other specimen and patient parameters. There are also several management reports defined to indicate which pathologists are using synoptic worksheets and for what types of cases; for cases with incomplete worksheets; and for searching cases by natural language or SNOMED coding to determine usage of worksheets.

## Results

The synoptic worksheets for Bone marrow malignancies, Non-Hodgkin's, Hodgkin and Gastrointestinal Lymphoma are implemented for use in our Laboratory Information System from February 1, 2006. These appear as an accession summary or synopsis within the final pathology report. In addition, the synoptic data can be exported to the research data base (hematolymphoid neoplasm virtual tissue repository) for linking pathology details of the banked tissues. To date there are a total of 223 cases of various hemopoietic and lymphoid neoplasms (Bone marrow malignancies, n = 28, Non-Hodgkin Lymphoma, n = 166, Hodgkin Lymphoma, n = 22, and Gastrointestinal Lymphoma, n = 07 cases) with completed synoptic worksheets. Synoptic Reporting, either as part of the pathology report or replacing the free text component has provided uniformity with standardized data elements in forms of checklists thus ensuring the pathologist makes note of these findings in their reports.

## Discussion

Traditionally, narrative descriptive reports have been used in surgical pathology to convey valuable diagnostic information that is predictive of the prognosis and biological behavior of a disease process. Such information is of immense value in making treatment decisions such as adjuvant therapy, radiation, chemotherapy and other interventions [1-7]. To enhance the clinical management of cancer, committees have been assembled by the American Cancer Society, American College of Surgeons, and American College of Pathologists to develop guidelines for data to be collected and included in routine pathology reports [6,9,10].

Traditional narrative and descriptive reports in free text format have significant variability because different pathologists use a multitude of different reporting styles to describe their findings. More often such variability results in pathology reports missing important clinically relevant data elements such as margins, lymphatic invasion etc. This ultimately gets reflected in an error making proper management plan for individual case.

Synoptic Reporting, either as part of the pathology report or replacing the free text component has uniformity with standardized data elements in forms of checklists thus ensuring the pathologist makes note of these findings in their reports [2-4,7,15]. In addition, synoptic data entry serves as methods of producing standardized reports, leading to improved pathology reports with the capacity for quality assurance and control. As clinicians rely on accurate and consistent diagnosis and staging information dictated by pathologists as basis to treatment recommendations and ultimate survival predictions, with the advent of checklist items, the answers would be more clear and consistent reducing the need to re-review slides. Furthermore, checklists could reduce time spent on signing out and reassessment such cases. Discrete checklist values can lend to improved assessment of quality of care studies, marketing and research activities [6].

The Cancer Registry can use the synoptic template to extract common data elements from a completed pathology report to populate the registry environment with subsequent linkage to the centralized integrated data annotation and query engine for subsequent research and sharing the data across. Synoptic reports can be utilized in the clinical environment and impact the work flow and serve as a conduit to extract information for translational cancer research. Currently, most Laboratory Information Systems do not support discrete data elements for synoptic data elements thus, the CAP protocols and checklists have been incorporated as unstructured text blocks which are embedded in the pathology reports. This arrangement results in the presentation of pertinent pathology data in a cumbersome and difficult to access format [6,8,15].

Text-only format provides less than optimal for entry of selected data elements and presentation on reports, and many obstacles to data retrieval by distinct data elements as audited in a project in early 2004. Therefore, data elements in text-based outlines against required and other data elements defined in CAP checklists needs to be converted to synoptic worksheets in CoPathPlus. At the University of Pittsburgh Medical Center, we have used a "synoptic reporting" tool (Cerner CoPathPlus) to incorporate the College of American Pathologists checklists as discrete data elements, allowing for storage of data elements in a relational table within the Laboratory Information System. These checklists have been modified into these synoptic worksheets for various organ systems and malignancies. We have also used CAP checklists created by Cerner DHT to supplement the library of checklists available for use in pathology reports [15].

Although the synoptic tool is an interesting and important way of conveying diagnostic and prognostic information to the clinicians, its use is controversial among pathologists. Based on our experience at our institute we encountered a range of responses when the synoptic tool was introduced. Some pathologists really liked while others resisted the use of the tool. We also learnt that after the tool was deployed for some time and pathologists became familiar with it, the degree of resistance towards the tool decreased.

There are various reasons why the synoptic tool may not be easily accepted by pathologists. Some pathologists consider this as a relatively cumbersome and time-consuming process, and require potential additional steps to enter and/or edit the worksheets compared to usual free text reports, therefore, limiting its universal acceptance. Additionally, this allows less flexibility for nuanced diagnoses or microscopic findings. It highly depends on the pathologist's experience, quality of training, and acceptance to this novel and intuitive tool, which would eventually impact consistency in the use of synoptic reporting. Additionally, this study describes our experience at our institute using a particular LIS for the creation and use of synoptic tools for hematopathology worksheets, the concept may be applied towards the development and implementation of similar worksheets using other LISs. In fact, since the deployment of our tools for cancer specimens, some other vendors have introduced similar tools for use in their particular LIS. As discussed in the manuscript, the synoptic tool allows for adaptability and flexibility by combining other patient information such as CBC, chemistry values etc in the same worksheets for displaying in the report and afterwards archiving in a relational database for mining for clinical and therapeutic decision-making. The intent of this study was to document the development and use of the tool for diagnosis of hematological and lymphoid neoplasms which we consider as an important first step. While, we hypothesize based on experience that the synoptic reporting is a superior method for entry and display of pathology information with fewer typographical errors as well as errors of omission, we have started initial studies to address these above-mentioned issues. We are in the process of carefully studying the turnaround times, typographical errors and other parameters comparing free text vs. standardized reports. This will involve collecting this type of a data over a long period of time, which will be communicated in a future publication. Our intention is to put our methodology and workflow out to the informatics and research community to start a dialogue about the standardized reporting process for hematological and lymphoid neoplasms.

## Conclusion

In conclusion, synoptic reporting provides a structured way of entering the diagnostic as well as prognostic information for a particular pathology specimen or sample, thereby reducing transcription services and reducing specimen turnaround time. Furthermore, it provides accurate and consistent diagnostic information dictated by pathologists as a basis for appropriate therapeutic modalities. Using synoptic reports, consistent data elements with minimized typographical and transcription errors can be generated and placed in the LIS relational database, enabling quicker access to desired information and improved communication for appropriate cancer management. Thus our goal is to provide templates that will serve as a conduit for capturing and storing data in the virtual biorepository for translational research, in addition to the diagnosis and management of the patient. Such uniformity of data lends itself to subsequent ease of data viewing and extraction, as demonstrated by rapid production of standardized, high-quality data from the hematological malignancy specimens. The more the standardized elements are, the higher the risk of 'doing something wrong', thus leading to possible legal actions. With the aid of CAP protocols and checklists, data of clinical importance can be provided in a fair and consistent manner for reporting. Furthermore, the "Global nature of cancer care" – cancer patients are being diagnosed and treated in a variety of settings requiring the need for uniform documentation of communication among health care facilities. Checklist items, consistent data elements and values would enable quicker access to desired information and improved communication for proper cancer management.

## Competing interests

The author(s) declare that they have no competing interests.

## Authors' contributions

SKM, AAP, SBW, MJB and AVP have contributed in study design, implementation and quality assurance of synoptic tool in the Laboratory Information System interface. ALP, LJD and GCW have contributed in the development and implementation of software tools for the synoptic interface. All authors contributed in implementation of standards and soft ware tools in synoptic reporting, drafting the manuscript, and read and approved the final document.

## Pre-publication history

The pre-publication history for this paper can be accessed here:


